# Maryland and District of Columbia Dental Association

**Published:** 1878-11

**Authors:** 


### ARTICLE II.

### *Maryland and District of Columbia Dental Association.*

[Reported for this Journal.]

The Fourth Annual Meeting of the Maryland and District of Columbia Dental Association was held in the City of Washington, on Tuesday, October 8th, in the parlor of the Imperial Hotel.

The President, Dr. B. F. Coy, presided, and called the

Association to order. A fair representation was present of Dentists from Baltimore and Washington, Dr. Atkinson of New York, and J. J. Caldwell, M. D., of Baltimore, and others from more distant cities.

A brief address of welcome was read by Dr. J. B. Ten Eyck, of Washington, D. C., which was followed by the annual address of the President, who alluded to the harmonious working of the Society in the past, and the fraternal feelings of all. The true work of the Society was just begun, and as we progress new fields of work show themselves. Whatever of interest that had transpired since the last meeting had been published in the Journals, and was doubtless attentively perused by all. The "New Departure" of Drs. Flagg, Chase and Palmer, was the most notable of the events of the year—an "old departure" it would doubtless be called by many; it was indeed only a statement in the main of old and well-known facts.

The division of the Association into "Sections" was a strong move toward organized work, and will greatly facilitate the studies of all in unknown or partially developed truths. Some improvements in the working of this plan will doubtless be made.

A question on which there seems a good deal of apathy, but which was one of paramount importance, was the prosecution of the New Rubber Suit. The gentleman who was now giving his sole attention and all his time toward this work should be amply repaid, and it should be the duty and pleasure of the Association to see that funds were provided to carry on this suit.

The presence of members of the Medical Profession among us was gratifying, and he trusted that we might be a mutual profit to each other.

After some routine business the Association adjourned.

### Afternoon.

The calling of Sections was taken up. The first Section, that of Anatomy and Physiology, was represented by Dr.

Atkinson, who, owing to late notification, had not prepared a paper or written report. He had a paper on Pathology and Therapeutics, and also one on late Discoveries in Histology.

"Surgical and Operative Dentistry" was called, and a paper read by Dr. R. B. Donaldson, in which the "New Departure" creed was discussed. He thought it imperfect because supported by a theory so far unproven. It would surely take a remarkably clear demonstration of the truth indeed, to make the profession give up the use of gold. Is it possible that for fifty years the practice of the dentists has all been a huge mistake? Surely not. As to the theory of chemical action, it has not been finally accepted by those best qualified to judge of its merit. Gold has been used so successfully to make it impossible for such a theory to be true. The currents claimed to exist are undemonstrated. There is no intention to detract from the promulgators of this theory. They are in deep earnest, and entitled to great credit for their labors. But when facts are pressed to support theories, or claims are made for facts which are no facts, we can allow for fair intentions and no more. Our hopes are so often excited by promises of success, which fail of fulfillment, and we lose time in retracing our steps; so caution comes to us. The "eclectic" is the only proper system, and gold was certainly used by those who practised this system, more largely than any other material. Extensive use of this, and observation had taught us conclusively that if the end in view is to save teeth, we can come nearer to this end by eclectic treatment than by any other. That none of the materials used are best in all cases, but that each is best in its place. That was a fallacy which dogmatically asserted that "any tooth that was worth filling at all, was worth filling with gold," nor can we save all the teeth coming to us. But we can save for years of usefulness shells of teeth, with plastic fillings, which it were folly to try to save with gold. Still, while gold is not the best in all cases, it is certainly the best in very many cases. It has always been known that plastic fillings were eminently useful, though the reading of his (Dr. Flagg's) paper would lead one to suppose that he had discovered it.

Is it a fact, as Dr. Flagg asserts, that in spite of the advance of dentistry in this country, our people are semi-edentulous, and that this is largely due to the use of gold? This will hardly be admitted by us. True, the land is flooded by artificial teeth, but those who wear them are those who never visit the dentist except to get teeth extracted and others inserted. The responsibility for this state of affairs rests not upon the dentists but upon the people themselves, who for fear of pain, or from love of money will not submit to operations which would save their natural organs. It is an outrage to saddle this state of affairs on a profession whose labors are most eminently successful. If people will visit the dens of quacks and charlatans you should not hold the better part of the profession responsible for this. We can only attempt to educate, but so long as people are gullible, they will be deluded by flaming advertisements.

It is not possible to save some teeth permanently; but most of those who make the artificial teeth never filled the natural organs these are to take the place of. And is it any wonder that Dr. Flagg abandons the use of gold when he asserted only a few years ago that he could fill a tooth with gold in two minutes? No doubt *he* can save more teeth with Amalgam.

How contradictory is all this. It cannot greatly benefit or injure the profession, but it may lead to investigations which will do good; but certain it is of the long list of plastic fillings in the market few are reliable and none so under all circumstances. No amalgam is or can be always reliable, and if we had a specimen which was just the thing, no dependence could be placed on the manufacturers to supply a constant and perfectly uniform article.

A PAPER ON LOOSE TEETH AND THEIR TREATMENT, was read, prepared by Dr. Merrill. It developed nothing specially new in treatment.

Dr. B. F. Coy, was the author of the next paper on The Six Year Molars—their Treatment, &c. He alluded to the doctrine that inheritance of the jaw of one parent and the teeth of another gave rise to frequent disarrangement of the dental arch, the common sense remedy for which was extraction, the attempt at enlarging the width of the arch in such cases being usually a failure. The idea was advanced that the human skeleton was gradually growing less in size, and if the teeth do not decrease in size, there is no remedy but extraction to make room for the proper arrangement. It is a rare case perhaps when all the ten anterior teeth cannot be saved, and as the six-year molar comes in at a time when there are many drawbacks to its complete development, when it does its work of mastication and can step aside for the twelve-year molar, it is wise to extract it, if manifestly deficient in quality, and if there are symptoms of a crowded arch. Five years usually close the career of these teeth, and they should be removed in time for the advent of the next molars, when they would take their places, and the way be left clear for the third molar in turn.

The writer was opposed as a rule to the system of spreading the arch, as frequent disfigurement was produced, and the teeth themselves injured by the appliances used, to say nothing of the pain and annoyance to the patient. In all cases we should by management preserve the front teeth without filling. As a rule it was wise to extract the six year old molar in nineteen cases out of twenty.

The discussion of the subject of the morning was continued.

Dr. Atkinson desired to be set on the record as characterizing the articles of Dr. Flagg, published in the *Cosmos* under the title Dental Pathology and Therapeutics, as "Drivelling Idiocy."

He credited Drs. Flagg, Chase and Palmer with trying to do something, but to try to pass this off for having *done* something—against such an attempt he respectfully entered his caveat. If we teach, teach one truth and stand on it, not on an assumption of knowledge. He would say with regard to the theory of mixing of races that he was not a believer in the doctrine of inherited jaws from one parent and teeth from another—the blending was complete. The whole being was half and half.

The papers of the morning were further discussed by Drs. Hunt and Winder, the latter concluded, after eight years close observation, that eight out of ten children inherited the teeth of the father. This was in opposition to the commonly accepted doctrine on this subject, and he was agreed with by Dr. Coy.

The discussion was continued by Dr. Hunt who stated that of one thousand crania examined by Dr. Winder, less than thirty had misplaced or carious teeth. He inquired what was the cause of this departure from normality, and thought it was our duty to search for those causes, that posterity might receive the benefit of our researches and studies.

Dr. Winder related an incident in the history of Dr. Riggs, relative to the question of the injuriousness of tobacco to the teeth. Dr. Riggs had written a good deal in the affirmative of this question, but had confessed to him (Dr. Winder) privately, that he did not believe in the doctrine of its ill effects, and stated that he kept a vial of tobacco-juice (under another name) in his office and used it as a local application. He had found it superior to anything he had ever tried as a gum wash.

Dr. Atkinson dissented from the views taken by Dr. Coy, that there was an inheritance of teeth from one parent and jaws from another. He was sure that there was perfect blending of types.

Dr. Ten Eyck enquired how long it was judicious to allow the six year molars to remain, where circumstances indicated their final loss, and was answered that about the eleventh year was the proper time for their removal.

Dr. Hodgkin dissented from Dr. Hunt's theory that we must endeavor to educate the people up to a standard of development, and thought that there was little probability of our ever making selective breeders of men and women. So long as passion ruled—and it had and always would rule men in the selection of mates—there was no chance that a broad-jawed man would hunt up a like jawed woman.

Dr. Caldwell said that all infirm races faded away before the Anglo-Saxon. The history of all the world showed this. Where intermarriage of races occurred with the Anglo-Saxon, the inferior race, with its traces perished.

The subject was passed.

Dr. Atkinson read a paper on Physiology, taking the ground of correspondences or analogies in the human body to things in nature. He made the brain to have its analogue in the breathing; the face corresponded to æsthetics; the sacrum to the animal tail of the lower orders; logic found its counterpart in the frame-work of the universe, and had its analogue in the skeleton; mathematics was related to the limbs,—legs, arms, fingers, toes, etc; the pelvis and nates the cosmology of the universe; the genitals belonged to physics; chemistry was all the ingredients of the body, etc.

The paper was discussed by Drs. Winder, Hodgkin, Donaldson, Hunt and others. Dr. Hodgkin seemed inclined to make light of the doctrines of the paper, while Dr. Hunt said that it was not wise to throw aside as useless, things we might not understand. Many things which sound to us like empty words, are afterwards found to be the deepest truths. The fault was with ourselves, not with the paper. Adjourned.

### Night Session.

The report of the Committe on Sections was read, and Dr. Noble explained that in the inception of the work it could not be expected that this plan would work smoothly, many not fully comprehending the arrangement. After further explanation of the plan, showing how it was thought that by each member taking a special subject and working over that year after year, better work would be done than if performed in the usual desultory manner.

The various Sections then proceeded to elect chairmen,

(which Dr. Atkinson insisted should be *Chairmans*,) the Sections standing as below.

Sec. 1.—*Anatomy and Physiology.*—R. B. Winder; W. H. Atkinson; J. W. Correll, M. D.; J. J. Caldwell, M. D.; J. R. Walker; J. J. Williams; W. W. Evans; J. L. Wolf; Thos. B. Evans, M. D.; H. M. Schooley.

Sec. 2.—*Histology and Microscopy.*—W. H. Atkinson; W. H. Dwinelle; J. R. Walker; H. H. Keech; F. F. Drew; Thos. B. Evans; R. B. Winder; H. M. Schooley: Ephraim Cutter, M. D.

Sec. 3.—*Pathology and Etiology.*—E. P. Keech; W. H. Atkinson; J. R. Walker; H. H. Keech; F F. Drew; J. B. Hodgkin.

Sec. 4.—*Diagnosis and Therapeutics.*—J. J. Caldwell, M. D.; W. H. Atkinson; H. B. Noble; H. C. Thompson; B. F. Coy; C. T. Brockett; J. M. Riggs.

Sec. 5.—*Chemistry and Materia Medica.*—C. E. Duck; R. Finley Hunt; S. M. Field; J. J. Caldwell, M. D.

Sec. 6.—*Metallurgy and Chemistry of the Metals.*—R. Finley Hunt; A. H. King; Henry Townsend; C. E. Duck; E. Howard; M. W. Foster; J. B. Hodgkin.

Sec. 7.—*Anæsthesia and Anæsthetics.*—H. C. Thompson; J. J. Caldwell; B. F. Coy; G. B. Welch; J. B. Hodgkin; Lawrence Turnbull.

Sec. 8.—*Surgical and Operative Dentistry.*—B. F. Coy; R. B. Donaldson; H. C. Thompson; J. Curtiss Smithe; H. B. Noble; J. B. Ten Eyck; Wm. Merrill; C. T. Brockett; J. Emory Scott.

Sec. 9.—*Artificial Dentistry.*—M. W. Foster; T. S. Waters; C. E. Duck; W. W. Evans; O. H. Brightwell; R. Finley Hunt; E. Howard; A. H. King; H. Townsend; J. Curtiss Smithe.

Sec. 10.—*Dental Education and Literature.*—H. B. Noble; W. H. Dwinelle; T. G. Loockerman; B. F. Coy; J. B. Hodgkin; T. S. Waters; R. B. Donaldson.

Dr. Atkinson then delivered a lecture on Histology, Production, Maintenance and Reproduction of Tissue, illustrated with diagrams of the chick in various stages of hatching. In the course of his discussion of the evolution of tooth structure, he showed diagrams illustrating lacunæ, or their analogues in the dentine, with fibres crossing from one to the other, presenting a reticulated appearance. He showed also what he styled protoplasmic strings in enamel, and also lacunæ. He thought there was no differentiated nerve in dentine; and that as teeth were liable to be set on edge after the loss of Nasmyth's membrane, that this was proof of the existence of fibres for the nourishment of enamel. He also took the ground, in opposition to the books, that there was no vein or artery in the pulp, but only a capillary network; and was understood to say that the pulp was nerve tissue. Adjourned.

### SECOND DAY.—Morning.

E. Cutter, M. D. of Boston, and L. Turnbull, M. D. of Philadelphia, were elected honorary members of the Association.

Letters from Drs. Dabney and Edwards, of Va., regretting inability to attend, were read, and also from Dr. Dwinelle, of New York.

The report of the Section on Anæsthesia, was read by Dr. H. C. Thompson, of Washington, D. C. It was a copious resume of the status of *Anæsthesia* and *Anæsthetics*.

The following is an abstract of a part of the paper:

"After some prefatory remarks on the differences in opinion on the part of the advocates of ether and chloroform, and the statement that their differences might be in part reconciled by the differences between ordinary civil hospital cases as found in the North, and operations in the field:

"It is asked, 'is the proper study of the field of anæsthesia the previous condition of constitution of the patient? Is it surgical shock which kills the patient, or is it the drug? Is some obscure change in the blood itself the cause of the anæsthesia, and of the accidental catastrophe, or is it that to mere incidents as to position, dress, the method of administration, we may greatly ascribe the untoward result?'

These are too grave questions to be flippantly handled or hastily decided, and we honor the men who, in a true scientific spirit undertake their cautious, slow solution."

Dr. Waterman brings to the aid of the pathologist, one of the newest in application of scientific instruments in nosological art—the spectroscope, and with all the ardor of an enthusiast, attempts the demonstration of the problems of the life and death state—anæsthesia, as seen under nitrous oxide, ether and chloroform. Special attention is given by him to the first-named gas, which his demonstration *seems* to prove to be of great danger, and which his clinical practice corroborates fully. To summarise:—Blood seen under the spectroscope gives certain characteristic lines or bands in the spectrum. These, as are all other lines in spectral analysis, are constant and definite. No other could be mistaken for blood, nor could this be ever mistaken for any other organic substance. Oxydized blood displays a different spectrum from deoxydized blood, and putrid blood shows a spectrum peculiar to itself, unmistakably marked. The deoxydized blood can be restored to a normal state spectroscopically, by reoxydization, and the changes that play through it are sharply defined and well known.

On the other hand blood which has been subject to the action of nitrous oxide gas, and blood which has become putrid, show similar lines in the spectrum, and no manipulation can develop in blood acted on by this gas other lines than this "death line" of putridity, or its semblance, spectroscopically considered, and the conclusion which is reached seems an inevitable one, viz., that the changes wrought in blood by the Protoxide of Nitrogen and the destructive changes in which putridity is set up, are identical. If there is fault with this theory, it must be set down to an imperfect understanding of the conditions, for the defendants of the spectroscope contend for its infallibility, and with justice doubtless. We must guard this point by the observation that whilst according to the instrument all that its most zealous advocates claim for it, we must bear in mind that its interpreters are human, with human frailties, the most fatal of which to severe scientific research is the tendency to "jump to conclusions." It was Agassiz who claimed that not one man out of thirty was competent to make the simplest observation ; and we know the fatality which besets the most judicial of us in being able to see only that which we are looking for.

The rationale of the action of nitrous oxide is certainly obscure. The hurried respiration, the cyanosis, the staring eyes, are fearfully suggestive of partial asphyxia, and certainly suggest imperfect decarbonization of the vital fluid. On the other hand, the rapid recovery, the occasional cheerfulness, the lack of loss of tone, and, in a word the usual speedy return of normal conditions, as a rule, seem to indicate that no great morbid change can have taken place in the blood, and that injury is not often apparent. The effect of these studies however, in lessening the indiscriminate administration of this gas is evident; and curiously, coincidently with these investigations the demand for it seems to slacken.

The studies of Dr. Waterman on chloroform and ether, spectroscopically, are of less moment as to satisfactory results. He considers as one of the dangers of chloroform, the retention of its vapor in the lungs, poisoning much as does carbonic acid gas, by displacement, its specific gravity in vapor being great; and he suggests as a remedy the inhalation of hot air.

The paper of Prof. Chisholm on "What anæsthetic shall we use?" is a special plea for chloroform, in which he takes the bold ground that the dangers to be apprehended from the administration of that drug are in an inverse proportion to the profoundness of the anæsthetic condition, and that reflex action is the cause of syncope and death. The ground is taken boldly that as surgical shock in *ante* anæsthetic days was the occasional cause of death, so *now* the same cause inheres, and as it was then manifestly due to reflex disturbance, that so now the same reflex action and danger are to be and can be averted by profoundness of anæsthesia. That weak lungs or heart are no contra-indication of chloroform, but that they even suggest a more vigorous use of the drug;* as full anæsthesia will tend to so overwhelm the medulla as to prevent reflex action.

This is bold doctrine certainly, and the conclusions, though based on extensive practice and copious statistics, are such as most of us would shrink from. Yet we can hardly tell how far this shrinking from an acceptance of such doctrine is the effect of the previous instillment of a contrary doctrine, so potent is education and precedent in moulding our ideas of the proper thing. It is the bold who ever succeed; and that death at the surgeon's hands is due to timidity arising from imperfect knowledge rather than from the boldness of complete mastery of the subject is more true than we perhaps care to acknowledge.

The horizontal position is insisted upon by Prof. Chisholm, as indeed it is by all who use anæsthetics, as a *sine-qua-non* to its safe administration; and previous abstinence as a matter of course. The pallor which has frightened many timid operators, is as he suggests only the precursor of emesis, which if occuring, should be watched, as fatal accidents seem to have resulted from the entrance of the ejected food into the trachea.

Death at the hands of the dentist is, relatively speaking, much more common than at the hands of the surgeon, when chloroform is used. The solution seems to be found in the fact that anæmia of the brain is induced by the drug, which assuredly depresses all the vital functions, and this æuemia inducing syncope, is again reflected on the heart, inducing paralysis of that organ; then, with the two legs of the great tripod of Bichat taken away, the catastrophe is precipitated. Hence decubitus must be insisted upon.

*This is suggestive of the commendation of Mr. Sam Weller, on hot punch, which he observes was the best thing in the world for warding off an attack of rheumatism; and that if it failed to prevent an attack of that malady, it was because the patient fell into the vulgar use of not taking enough of it.

The preparation for anæsthesia is well-known. The horizontal position just alluded to, and the empty stomach; the perfect freedom of respiration, guaranteed by the loose clothing; and finally the administration of whiskey. This last should be ever borne in mind, remembering that alcohol is of itself a true anæsthetic, and its usefulness in conjunction with chloroform is no longer a matter of theory. Yet the old observation of Paget corroborated by every day experience must be borne ever in mind, that none bear operations so badly,—in no case does the surgeon so shrink from an operation, as on the confirmed inebriate.

Lately, Dr. Hunter Maguire, of Richmond, has announced, and his observations are confirmed by others, that Quinia is of essential service in preventing or modifying surgical shock, often more to be dreaded than the effects of the anæsthetic. A discovery of propound importance, if true; and one which will go far to relieve the mind of the sympathetic and ever solicitious surgeon.

Dr. Combes, of Louisville, Ky., in a late paper on chloroform, thinks the chief danger from that agent to arise from an accumulation of it in the system, which result he judges more likely to occur where it is given tardidly. And the weight of opinion is at present in favor of rapid overwhelming of the patient, rather than the slow and cautious use of the drug formerly advised. Caution is not at all incompatible with vigorous action, and the timid creeping of the fearful may indeed be a cause of unsuspected danger —injury arising out of the very means taken to prevent it. Dr. Combes thinks our American people less easily overcome by chloroform than Europeans, except the Irish, and finds that Germans and Negroes yield readily to its influence.

We may sum up the present attitude of the advocates of anæsthesia in the famous words of the famous Syme, who said that he "had given chloroform for years without an accident. He attributed his success to two causes: first, he always used pure chloroform, and second, he gave plenty of it." And he is followed by Prof. Chisolm with the glowing tribute to chloroform, that it is God's latest and best gift to man.

[TO BE CONTINUED.]

